# Efficacy and safety of dual intravenous artesunate plus quinine compared to intravenous artesunate for cerebral malaria in a triple blinded parallel multisite randomized controlled trial in Nigerian children: DUAL PAQ TRIAL Protocol

**DOI:** 10.1186/s13063-021-05634-6

**Published:** 2021-10-20

**Authors:** Michael Abel Alao, Adebola Emmanuel Orimadegun, Olayinka Rasheed Ibrahim, Abayomi O. Oyenuga, Adanze Onyenonachi Asinobi, Daniel Adedosu Gbadero, Ifeoma Joy Okoye, Emmanuel Okechukwu Nna

**Affiliations:** 1 Department of Paediatric, University College Hospital, University of Ibadan, Ibadan, Nigeria; 2 College of Medicine, University of Ibadan, Ibadan, Nigeria; 3 Institute of Child Health, University of Ibadan, Ibadan, Nigeria; 4 Department of Paediatrics, Federal Medical Centre, Lagos, Katsina Nigeria; 5 University of Minnesota, Minneapolis, MN USA; 6 Department of Radiation Medicine, University of Nigeria Teaching Hospital, Enugu, Nigeria; 7 The Molecular Pathology Institute, 44 Rangers Avenue, Independence Layout, Enugu, Nigeria

**Keywords:** Cerebral malaria, Artesunate, Quinine, Randomized controlled trial, Blinding

## Abstract

**Background:**

Evidence exists as to the criticality of the first 24 h in the management of cerebral malaria. The morbidity and the mortality rate (35%) with the current intravenous monotherapy for the initial treatment of cerebral malaria are unacceptably high. Combination therapy and a shorter course of effective medication have been shown to improve outcomes in human participants in the treatment of other diseases. This study outlines a protocol to conduct a triple blinded parallel randomized controlled trial on cerebral malaria using dual intravenous medications compared to the current standard of monotherapy.

**Methods:**

This is a parallel multi-site randomized controlled superiority triple blinded trial consisting of intravenous artesunate plus quinine and a control arm of intravenous artesunate only. Eligible and assenting children aged 6 months to 17 years will be recruited from 4 tertiary hospitals by random selection from the list of tertiary hospitals in Nigeria. Participants will be randomized and assigned in parallel into two arms using random numbers generated from GraphPad Prism (version 9) by a clinical pharmacologist who has no link with the investigators, the patients, or the statistician. The primary measurable outcome is survival at 12, 24, and 48 h post-randomization. A composite secondary outcome consists of the number of children that regained consciousness, parasitaemia and defervescence at 12 and 24 h post-randomization and haematological and inflammatory markers at 24 and 48 h post-randomization. Adverse events both solicited and unsolicited are recorded all through the study post-randomization. The study is approved by the State Research Ethics Review Committee. Data analysis will be performed in GraphPad Prism version 9.

**Discussion:**

The outcome of this analysis will give insight into the efficacy and safety of dual intravenous antimalaria in the treatment of cerebral malaria among Nigerian children compared with the standard of care. The safety profile of this intervention will also be highlighted. This may help inform physicians on the optimal treatment for cerebral malaria to improve outcomes and reduce recrudescence and treatment failure.

**Trial registration:**

Pan Africa Clinical Trial Registry PACTR202102893629864. 23/02/2021.

**Supplementary Information:**

The online version contains supplementary material available at 10.1186/s13063-021-05634-6.

## Administrative information

Administrative details of DUAL PAQ.

**Table:** 

Title {1}	Efficacy and safety of dual intravenous artesunate plus quinine compared to intravenous artesunate for cerebral malaria in a triple blinded parallel multisite randomized controlled trial in Nigerian children: DUAL PAQ TRIAL Protocol
Trial registration {2a and 2b}.	This study was registered at Pan Africa Clinical Trial Registry no PACTR202102893629864
Protocol version {3}	2018-05-15 version 1
Funding {4}	This study will be funded by the authors.
Author details {5a}	1. **Michael Abel Alao:** Department of Paediatric, University College Hospital and College of Medicine, University of Ibadan 2. **Adebola Emmanuel Orimadegun:** Institute of Child Health, University of Ibadan 3. **Olayinka Rasheed Ibrahim:** Department of Paediatrics, Federal Medical Centre, Kastina, Kastina State, Nigeria 4. **Abayomi Oyeniyi Oyenuga:** University of Minnesota: Minneapolis, Minnesota, US 5. **Adanze Onyenonachi Asinobi:** Department of Paediatrics University College Hospital Ibadan 6. **Daniel Adedosu Gbadero:** Department of Paediatrics, Bowen University Teaching Hospital Ogbomoso 7. **Ifeoma Joy Okoye:** Department of Radiation Medicine, University of Nigeria Teaching Hospital, Enugu 8. **Emmanuel Okechukwu Nna:** The Molecular Pathology Institute 44 Rangers Avenue, Independence Layout, Enugu, Enugu State
Name and contact information for the trial sponsor {5b}	Michael Abel Alao, Department of Paediatrics, Bowen University Teaching Hospital, Box 15, Ogbomoso, Oyo State, Nigeria. Email: mikevikefountains@gmail.com Tel: +2348053967839 **ORCID ID** Michael Abel Alao https://orcid.org/0000-0003-0109-4435
Role of sponsor {5c}	**The sponsor would serve as funders.He is involved as a member of the steering committee. He designed the study, will be involved with interpretation of result; writing of the report; and the decision to submit the report for publication, and data availability**

## Background and rationale {6a}

Malaria remains a global health problem and a threat to human survival in over one hundred countries of the world [1–6]. It is one of the leading causes of morbidity and mortality among the under-five in sub-Saharan Africa [1]. In spite of the concerted efforts and huge resources dedicated to malaria eradication, its tales of woes remains strong and visible [1, 3, 7]. The socio-economic burden of malaria in endemic communities, especially in sub-Saharan Africa, is still colossal. It is a major source of drain to the scarce resources in low- and middle-income countries (LMIC) of the world [7].

Reports from previous studies [1, 2, 7] have shown that severe/complicated malaria carries 100% mortality if untreated. The most critical moment determining outcomes of severe malaria is the first 24 h from disease onset, often characterized by multiple organ dysfunction syndrome [1, 2]. However, current treatment regimens use monotherapy that provides an opportunity for the development of drug resistance and sub-optimal outcome [7]. Current mortality from severe malaria ranges between 22.5 and 34.7% in Africa and Asia, respectively [1, 8–10]. Although there has been some improvement in outcomes [1, 8, 11], the high morbidity and mortality in the endemic countries are still concerning.

The current treatment for uncomplicated malaria advocates artemisinin-based combination therapy. However, the question is whether the use of dual artemisinin-based intravenous antimalarials is more efficacious than the current monotherapy. Indices of comparison consist of survival, recovery of consciousness, parasite clearance, fever resolution and adverse events in cerebral malaria. We hypothesize that initiation of intravenous artesunate plus quinine within the first 24 h of cerebral malaria will be more efficacious than intravenous artesunate only [9, 10]. This protocol describes a parallel multisite randomized triple blinded controlled trial to compare intravenous artesunate plus quinine with artesunate only in Nigerian children aged 6 months to 17 years.

## Definition of terms

Cerebral malaria (WHO guidelines for malaria, 2021) is severe *Plasmodium falciparum* malaria with impaired consciousness (Glasgow Coma Scale < 11, Blantyre Coma Scale < 3) persisting for > 1 h after a seizure.

Impaired consciousness is a Glasgow Coma Score < 11 in adults or a Blantyre Coma Score < 3 in children.

## Research problem

Severe malaria with multiple organ dysfunction is a major killer of children under-five in sub-Saharan Africa [1]. The morbidity and mortality rate of 35% with the current monotherapy is unacceptably high [8]. There is a need for modification of the current treatment regimen to improve outcomes and prevent the development of resistance, recrudescence and treatment failure.

## Rationale for the study

Combination therapy and a shorter course of effective medication in the treatment of diseases have been shown to improve outcomes in human immunodeficiency virus AID (HIV-AID) infection, tuberculosis, acute pyogenic infections and epilepsy [12–15]. If the critical time for severe malaria treatment is the first 24 h of illness, aggressive therapy targeted during this time using a synergistic combination of intravenous artesunate-quinine has a potential for rapid parasite clearance and reduce morbidity and mortality. This trial is feasible because of the long-standing biosafety profiles of artesunate and quinine. We are optimistic that the results of this trial may stimulate the interest of pharmaceutical companies in the development of newer formulations of intravenous antimalarial combination therapies; this could improve the availability and administration of treatment within the first 24 h and consequently improve the health outcomes in patients diagnosed with cerebral malaria.

## Objectives {7}

### General objective

The general objective is to determine the efficacy and safety of dual intravenous artesunate plus quinine combination regimen against the monotherapy of artesunate in the treatment of cerebral malaria.

### Specific objectives

The specific objectives are to determine the following:

- Survival rate from cerebral malaria on dual intravenous artesunate plus quinine compared to intravenous artesunate only
- The parasite clearance rate measured by microscopy
- Fever defervescence, measured by axillary temperature
- Time to recovery of consciousness
- Time to the conversion of dual intravenous treatment to a full dose of enteral artemisinin-based combination therapy
- Side effect profile of interventions (treatment and control arms)
- Neurologic status at the fourth week

## Trial design {8}

This will be a triple blinded, parallel randomized controlled trial consisting of one treatment (experimental arm: intravenous artesunate + quinine) and one control arm (intravenous artesunate). Patient allocation will be in the ratio of 1:1. The assignment of patients into the study arms will be randomized.

## Methods: participants, interventions, and outcomes

**Fig. 1:**
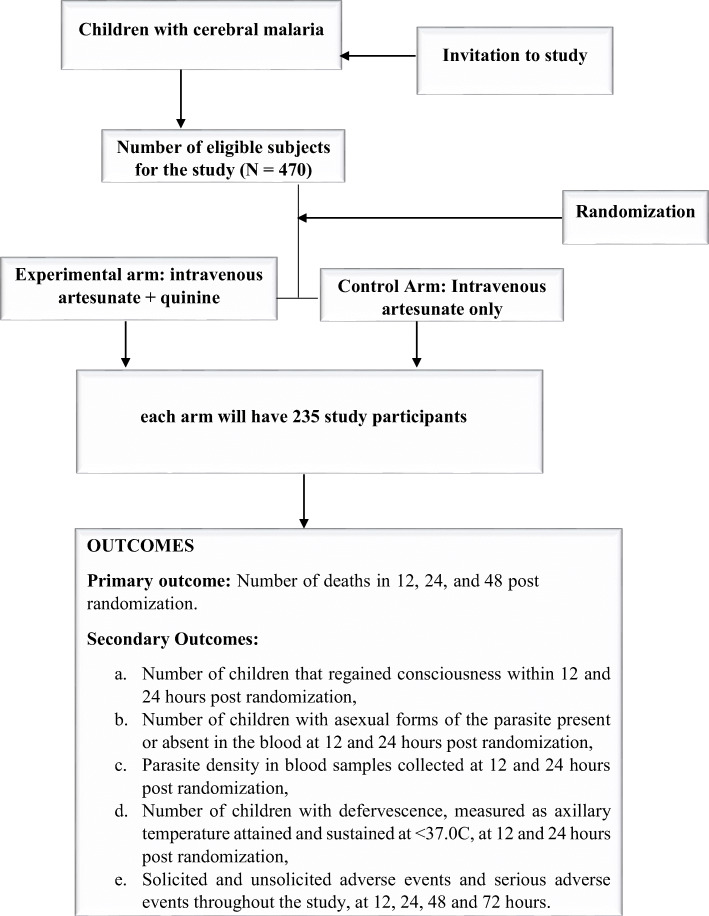
Participant flow chart

The participants’ flow chart from invitation through enrolment, allocation, and assessment is shown in Fig. 1

### Study setting {9}

This study is a multi-centre RCT involving 4 tertiary hospitals (one private and three public) located in three of the six geopolitical regions in Nigeria [16, 17]. The selected tertiary hospital includes University College Hospital, Ibadan; Bowen University Teaching Hospital, Ogbomoso; the Federal Medical Centre Katsina, Katsina State; and the University of Maiduguri Teaching Hospital, Maiduguri.

**Fig. 2:**
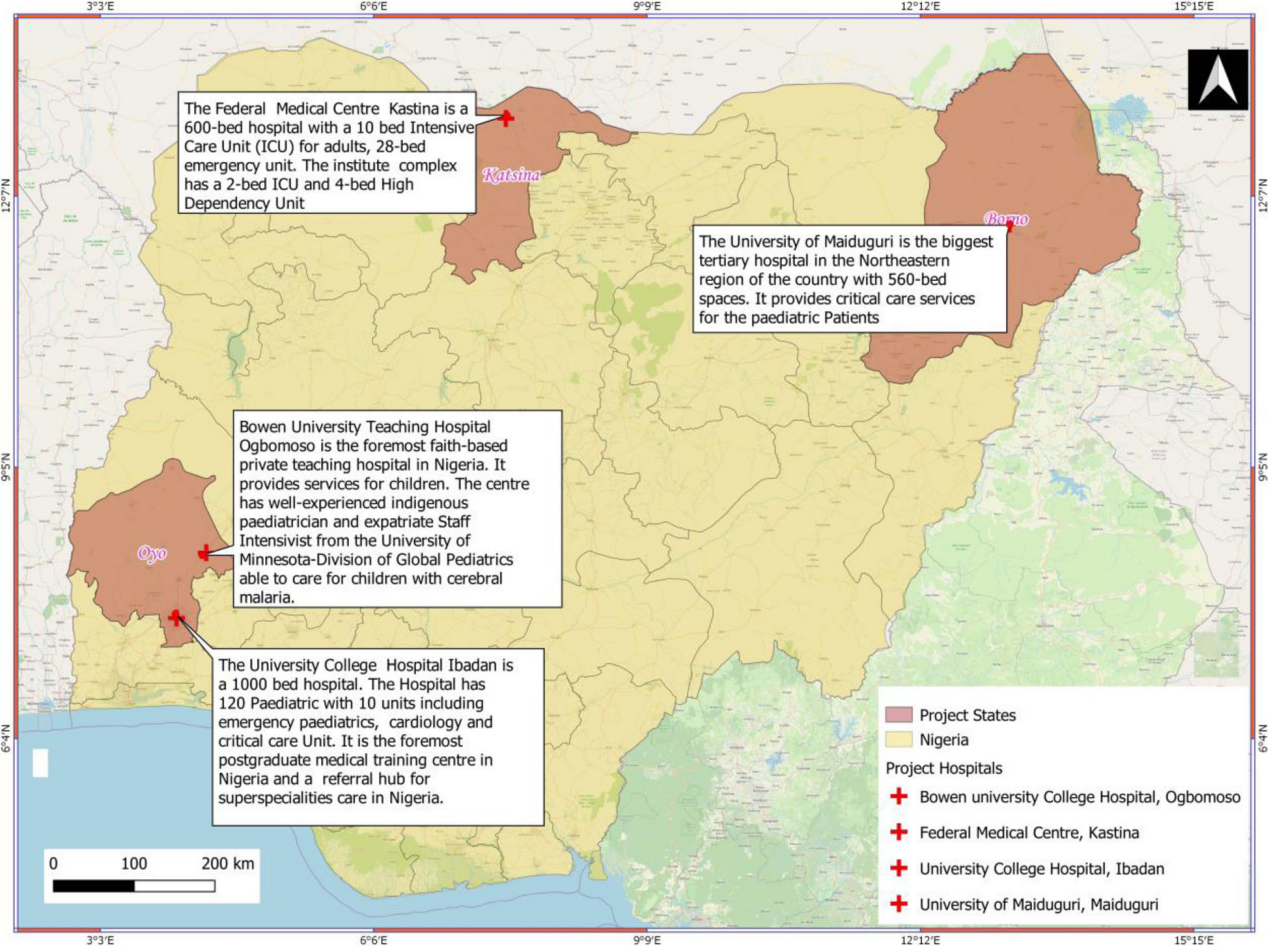
Trial site locations

All the facilities have the human capacity to manage critically ill children. Other details are given: administrative details (the ‘Administrative information’ section) and trial site locations (Fig. 2).

### Eligibility criteria {10}

#### Inclusion criteria

1. Children between the ages of 6 months and 17 years as at the last birthday.
2. Children with impaired consciousness (Glasgow Coma Scale < 11 for children older than 5 years or Blantyre Coma Scale < 3 children younger than 5 years) persisting for > 1 h after a seizure in the presence of demonstrable peripheral asexual *P. falciparum* parasitaemia. This is in line with the WHO guideline for malaria, 2021.

#### Exclusion criteria

1. Children with mixed species of *Plasmodium* infection
2. Prior treatment with artemisinin derivatives or quinine or other antimicrobial with antimalarial activities including other antimalaria or antibiotics within 12 h of screening
3. Children with positive biochemical and/or microbiological cerebrospinal fluid analysis result consistent with a diagnosis of meningitis
4. Children who have intracranial infections or intracranial space-occupying lesions
5. Children with previous neurological deficit either focal or global
6. Children whose caregivers declined granting consent for their participation in the study

### Who will take informed consent? {26a}

Prior to recruitment, the research will be explained to the parents/caregivers by the investigators who will obtain written informed consent. Parents/caregivers will be informed of their freedom to refuse to take part in the study without any negative consequences to them or their wards in the course of the treatment.

### Additional consent provisions for collection and use of participant data and biological specimens {26b}

Additional consent will be obtained for data availability for secondary analysis and for ancillary studies on biological specimens collected for the research.

### Interventions

#### Intervention description {11a}

The experimental arm will receive a combination of intravenous quinine plus artesunate. A clinical pharmacologist (unblinded) will deliver the allocated treatment to the clinician (blinded) for administration.

This group will receive a combination of intravenous quinine plus artesunate. The quinine will be given at a loading dose of 20 mg/kg and subsequently 10 mg/kg 8 h for 72 h. However, if the patient remains unconscious, the dose will be reduced to 7.5 mg/kg 8 hourly until he/she regains consciousness. In parallel, intravenous artesunate at 3 mg/kg if weight is less than 20 kg or 2.4 mg/kg if weight is greater than 20 kg at 0, 12, and 24 h and subsequently a daily dose of 1.2 mg/kg/day until conscious.

#### Explanation for the choice of comparators {6b}

Intravenous artesunate alone will be used as a comparator in this study. The choice of the two antimalaria is based on the difference in pharmacodynamics and varying mechanism of actions of the artemisinin derivatives compared with quinine. It is assumed that a synergistic action of the combined drugs against the erythrocytic and gametophyte stages of malaria parasite may enhance clearance. In addition, quinine is the only licenced alternative intravenous antimalaria by the WHO besides the artemisinin derivatives. These are the current standard treatments for cerebral malaria by the World Health Organization. In order to facilitate blinding, the monotherapy group (control) will also receive intravenous dextrose water or water for injection to maintain the similarity of treatment administration with the dual-therapy group (the treatment arm).

#### Control arm: intravenous artesunate only

This arm will receive intravenous artesunate at 3 mg/kg if weight is less than 20 kg or 2.4 mg/kg if weight is greater than 20 kg at 0, 12, and 24 h and subsequently a daily dose of 1.2 mg/kg/day until conscious. Additionally, intravenous 10% dextrose water will be given in lieu of intravenous quinine.

#### All the groups

The two groups will receive enteral artemisinin-based combination therapy (ACT) as soon as they regain consciousness and can tolerate the full course of oral ACT in line with WHO recommendation.

#### Criteria for discontinuing or modifying allocated interventions {11b}

1. Participants who regained full recovery after initial treatment.
2. Participants who also wish to exit after due counselling.
3. Participants with intractable hypoglycaemia despite maintenance at 12.5% glucose.
4. Participants with dysrhythmias will be unblinded.
5. Participants who develop commodities of severe malaria that could confound the measurable outcomes such as uraemic encephalopathy.
6. Participant with a new disease besides cerebral malaria or updated clinical history related to exclusion criteria.

#### Strategies to improve adherence to interventions {11c}

- Prior to recruitment, parents/caregivers of study participants will be educated on the complication of severe malaria to gain their support in the research.
- All study participants will enjoy social support throughout the hospital stay.
- The possible side effect profile of artesunate and quinine will be discussed with the parents/caregivers at the enrolments for the study.
- The study will only be responsible for the trial cost. This would include the cost of medications for the intervention, cost of managing adverse events from the intervention and the primary laboratory tests needed for the diagnosis of the disease in line with research ethics. Other costs will be the responsibility of the caregivers of the patients.

#### Relevant concomitant care permitted or prohibited during the trial {11d}

1. Concomitant treatment for septicaemia, severe anaemia, AKI stages 1 and 2 and hypoglycaemia will be permitted.
2. Treatments for other comorbidities of severe malaria which do not confound the protocol and measured outcomes will be permissible.

#### Provisions for post-trial care {30}

The participants with neurologic deficit as a result of the disease will be followed up by the paediatric neurologist for a continuum of care.

### Outcomes {12}

#### The primary outcome

1. The primary outcome is survival measured at 48 h post-randomization.

#### Secondary outcomes

- Number of patients that regained full consciousness as measured by GCS/MGCS of > 11 for children > 5 years or Blantyre Coma Scale of > 3 in children < 5 years at 12 and 24 h post-randomization.
- Number of patients with asexual form of malaria parasite present or absent at 12 and 24 h post-randomization.
- Number of patients that achieved fever defervescence (attainment and sustenance of axillary temperature of < 37.5 °C) at 12 and 24 h post-randomization.
- Parasite density at 12 and 24 h post-randomization.
- Number of patients with neurologic deficits at the fourth week (day 29) post-intervention in each of the arms. Neurology deficit is defined as the presence of impaired cognitive, motor or cranial nerve palsy and or persistence of convulsions.
- Time to convert to a full dose of enteral artemisinin-based combination therapy.
- The side effect profile (adverse events and serious adverse events) of medication in each arm of study: (i) solicited side effects include hypoglycaemia, arrhythmia, thrombocytopenia, tinnitus and anaemia; (ii) unsolicited adverse events as reported by patients post-randomization; (iii) random plasma glucose will be measured 6 hourly until 48 h after parasite clearance. Hypoglycaemia will be corrected with 2-4 ml/kg of 10% D/W and maintenance with glucose infusion at 6–8 mg/kg/min until euglycaemic; (iv) electrocardiography will be recorded before the administration of anti-malaria and 4 h after an intervention. The QT and RR will be measured by software and checked manually. Bazett’s formula will be used to correct the QT interval (QTc = QT/RR**0.5). Prolonged QT interval managed as per hospital protocol; (v) haematocrit, serum ferritin, platelet count and erythrocyte sedimentation rate will be monitored at baseline and 24 h and 48 h post-randomization.
- Plasma levels of inflammatory markers including C reactive protein, procalcitonin, lactate dehydrogenase and interleukin 6 at baseline, 24 h, and 48 h. Inflammatory markers and cytokine are included to assess a possible ‘cytokine storm’ in the course of parasite clearance.

### Participant timeline {13} [18]

**Table:** 

	Study period						
	Timeline						
**Event/activities**	**0 h**	**12 h**	**24 h**	**48 h**	**72 h**	**29 days**	***t***_***x***_
**Enrolment**							
Eligibility screen	x						
Informed consent	x						
*Anthropometry measurement*	x						
*Clinical history and detailed CNS examination*	x					X	
Allocation	X						
**Interventions**							
*Artesunate + quinine—8 hourly*	x	x	x	x	x	…	
*Artesunate—12 hourly*	x	x	x	x	x	…	
**Assessments**							
Electrocardiograhy	x	x	x	x	x		
*Survival*		x	x	x	x		
*Regained consciousness*		x	X				
*Parasitaemia*		X	X			.	
*Defervescence*		X	X				
*Adverse events*	x	x	x	x	x	x	x
*Haematological indices*	x		x	x			
*Inflammatory markers*	x		x	x			
*Glucose monitoring*	x	x	x	x	x	x	

### Sample size {14}

The sample size for this study was calculated using the G*Power calculator, based on the reported outcomes of cerebral malaria with quinine monotherapy (23.0%) and a dual quinine and artesunate combination therapy (12.5%) [10, 18–20]. Going for a difference of 23.0 to 12.5%, a sample size of 470 participants is required accounting for a 10% attrition rate at 80% power of a 2-sided alpha level of significance of 0.05. Based on 1:1 allocation, the treatment arm will have 235 participants and the same number for the control arm. The details of the selected studies for the sample size calculation are shown in a supplementary document as a table.

#### Invitation

The caregivers/parents of the eligible patients will be verbally invited during their hospital admission. The participant information sheet (PIS) will be explained to them in the language they understand.

#### Eligibility

Participants will be assessed based on the eligibility criteria enumerated above.

#### Enrolment

Eligible participants will be enrolled in the study after giving a written informed consent.

#### Informed consent

Parents and caregivers will give a written informed consent during enrolment. This will be signed by the principal investigator or co-investigator, the parent/caregiver and a witness. A copy of the informed consent will be retained by the parent, while a copy will be kept in the patient’s file.

### Sampling technique and assignment of interventions: allocation

An allocation sequence for the 2 arms will be generated for eligible patients using simple randomization from GraphPad Prism version 9. In the selected hospitals, patients with cerebral malaria, confirmed by a blood film malaria test (microscopy) but with a negative cerebrospinal fluid analysis result whose parents/caregivers give informed consent, will be assigned to one of the 2 arms based on the assignment sequence.

### Sequence generation {16a}

Sequence generation and patient’s allocation to either an experimental arm of intravenous artesunate and quinine and a control arm of intravenous artesunate and intravenous 10% dextrose water by the centrally located clinical pharmacologist (unblinded) who has no link with the other research team.

### Concealment mechanism {16b}

Participants’ allocation to any of the treatment arms will be concealed from the investigators, the patients, and the statistician (triple blinding) in an opaque envelope. The clinical pharmacologist will deliver an allocated intervention consisting of a box with a unique identification number linked to the sequentially generated number. The box would contain the study drug, case record form, and all the disposable required for drug administration and blood sampling by the physician. The packaging for the drugs and the dextrose water shams will be similar for both the treatment arm and the control. Centres will call the central pharmacologist to get concealed numbers at the time of enrolling a patient. When a patient is enrolled, one of the sealed envelopes will be opened for the allocation.

### Implementation {16c}

A clinical pharmacologist will implement randomization and treatment allocation for the multisite study with the aid of interactive voice response system interventions.

### Assignment of interventions: blinding

#### Who will be blinded {17a}

The treatment will be triple blinded. The investigator/clinician, parents/caregivers, and the consultant paediatrician (outcome assessor) will not be aware (blinded) of the interventions.

#### Procedure for unblinding if needed {17b}

When there is a serious adverse event that is life-threatening or features of quinine toxicity, the clinical pharmacologist will be called to unblind the allocation since he is the only one who has access to the concealed allocation.

### Data collection and management

#### Plans for assessment and collection of outcomes {18a}

Prior to the commencement of the trial, a pilot study of 20 patients will be conducted at the University College Hospital Ibadan site only, where the steering committee members are alongside the focal leads from the other three collaborating centres in order to test the questionnaire and the protocol. Data from the pilot study will be entered into a Microsoft Excel spreadsheet uploaded into GraphPad Prism version 9 and checked for appropriateness, completeness, and adequacy. Observation from the dummy analysis will be used to improve the data entry and address other observed limitations.

A debriefing session will be held to review the research process and appropriate adjustments where needed to improve the quality of the instrument and enhance understanding of the protocol for a better outcome. Subsequently, the site principal investigators will step down such training to the collaborating centres.

#### Plans to promote participant retention and complete follow-up {18b}

The study participants will be contacted via their phone to remind them of the next visit to the neurologist. The transportation fare will be borne by the research sponsor to facilitate retention on follow-up.

#### Data management {19}

Data obtained from the study will be entered into a password protected and encrypted institutional REDCap database. Only specific individuals from the collaborating centres will be given access to the database. All data from all the centres will be de-identified and managed through secure code. The data assessor (statistician) will be blinded to the data until the final stage of analysis.

#### Confidentiality {27}

All information collected in this study will be given code numbers, and no name will be recorded. This cannot be linked to the patients, parents, or care providers in any way. Identifiers will not be used in any publication or reports from the study.

#### Plans for collection, laboratory evaluation, and storage of biological specimens for genetic or molecular analysis in this trial/future use {33}

This trial has no intention for a genetic study on the respondent.

### Statistical methods for primary and secondary outcomes {20a}

Data from this study will be analysed using the GraphPad Prism 9 (GraphPad Software, 2365 Northside Dr. Suite 560, San Diego, CA, 92108). The appropriate descriptive statistics will be used to present the socio-demographic characteristics of study participants. The comparison of categorical outcomes between the arms will be analysed using the chi-squared or Fisher’s exact tests, as appropriate, and presented as risk differences, risk ratios, and 95% confidence intervals. Kaplan-Meier analysis and Cox regression models will be used to compare the recovery of consciousness, defervescence, and survival.

### Interim analyses {21b}

A blinded interim analysis will be conducted at 30% of enrollment, of which 141 participants would be available for efficacy comparison (the number of survival in cerebral malaria in each arm of the study). Using a conservative Haybiltte-Peto [21] repeated assessments criterion, the stopping early will be recommended at *p* < 0.001 and final significance at *p* < 0.05.

The results will be made accessible to the pharmacologist (unblinded) and the Data Safety and Monitoring Board (DSMB). Early stopping consideration will be on safety concerns to adverse events more than 50% in the study.

## Methods for additional analyses {20b}

Subgroup analysis will be performed using variables such as geopolitical region, gender, socio-economic status of parents and care provider, level of education, and occupation. The primary outcome, survival in each study arm, will be analysed in each subgroup analysis.

### Methods in analysis to handle protocol non-adherence and any statistical methods to handle missing data {20c}

The result of the study will be analysed per protocol. The number of participants with missing data will be clearly stated, and the missing items will be mentioned.

### Plans to give access to the full protocol, participant-level data, and statistical code {31c}

The protocol shall be published in a peer-reviewed journal and made publicly accessible to interested individuals or bodies. The participant-level dataset and statistical code shall be made available after following due process adhering to good ethical standards.

### Oversight and monitoring

#### Composition of the coordinating centre and trial steering committee {5d}

A trial steering committee will consist of the principal investigator, two scientific enquiries, the public enquirer, and a biostatistician. They will meet frequently to provide an oversight function for the trial conduct over the four centres in the country.

Each centre will have a hospital trial group headed by a consultant paediatrician who will be saddled with running daily events in the hospital, providing organizational support and reporting on a weekly basis to the steering committee. All investigators and monitors will be GCP compliant.

#### Composition of the data monitoring committee, its role, and reporting structure {21a}

The Data Safety and Monitoring Board DSMB will consist of a multi-disciplinary team of five professionals from the University College Hospital Ibadan. They will have oversight monitoring and safety checks on the data quality and interim analysis of the trial data. They will ensure conformity to the trial protocol. In the event of the inferiority of the primary and secondary outcomes of the trial compared with the standard of care and in the event of worse adverse reactions in the experimental arm, the trial would rely upon the extensive data safety review of the DSMB and the clinical pharmacologist to evoke a stopping rule on the trial.

#### Adverse event reporting and harms {22}

All participants in this trial will be monitored for medication side effects and adverse events. An adverse event (AE) is any untoward medical occurrence which happens to a participant on a trial. All AEs will be reported in the case report form (CRF) regardless of suspected causality. Medidata rave will be used for reporting of AEs. Serious adverse events (SAEs) include fatal medical occurrences, life-threatening, that require or prolong hospitalization or cause congenital anomaly or permanent or significant disability or incapacity. All SAEs will be reported to the sponsor, Local Health Research Ethics and DSMB, and Trial Steering Committee within 24 h.

#### Frequency and plans for auditing trial conduct {23}

The local ethics board will monitor the progress of this trial, and all medications and updates will be relayed to the body as events unfold. There will be weekly monitoring of trial sites.

#### Plans for communicating important protocol amendments to relevant parties {25}

Any modification to the protocol or trial update will be communicated to the ethical approval bodies, trial registry, and any other relevant parties within 24 h.

#### Dissemination plans {31a}

The outcome of this study will be communicated to participants, ethics board, and healthcare professionals. It will be published in peer-reviewed scientific journals for public access. Data will be made available to the public maintaining ethical guidance.

## Discussion

The outcome of this analysis will give insight into the efficacy and safety of dual intravenous artesunate plus quinine compared to artesunate only in the treatment of cerebral malaria in Nigerian children. The safety profile of this intervention will also be highlighted. This may help inform physicians on a better option for the treatment of cerebral malaria to improve outcomes; prevent the development of resistance, recrudescence, and treatment failure; and stimulate the interest of pharmaceutical companies in the development of newer formulations of dual intravenous antimalarial combination therapies.

## Trial status

The trial is active but not recruiting.

## Supplementary Information

**Additional file 1: Table S1.** The supplementary shows published data for the outcomes for interventions for cerebral malaria. Outcome for intravenous quinine alone was 23% to 26% while combines intravenous quinine and artesunate is 12.5%. Sinclair D, Donegan S, Isba R, Lalloo DG: Artesunate versus quinine for treating severe malaria. The Cochrane Library. 2012, 10.1002/14651858.CD005967.pub4.^a^ Hien TT, Arnold K, Vinh H, et al Comparison of artemisinin suppositories with intravenous artesunate and intravenous quinine in the treatment of cerebral malaria. Trans R Soc Trop Med Hyg 1992; 86: 582–83.^b^ Bartoloni A, Tomasoni L, Bartalesi F, Sani S, Zammarchi L, Castelli F, et al. Combined intravenous treatment with artesunate and quinine for severe malaria in Italy. Am J Trop Med Hyg 2010; 83: 274–76.^c^

## Abbreviations

RCT: Randomized controlled trial
PACTR: Pan Africa Clinical Trial Registry
GCMS: Glasgow Coma Score
GCP: Good Clinical Practice
MGCS: Modified Glasgow Coma Score
LMIC: Low- and middle-income countries
HIV: Human immunodeficiency syndrome
AIDS: Acquired immunodeficiency syndrome
