# Supplementary material for: Efficacy and safety of dual intravenous artesunate plus quinine compared to intravenous artesunate for cerebral malaria in a triple blinded parallel multisite randomized controlled trial in Nigerian children: DUAL PAQ TRIAL Protocol
Source: Trials. 2021 Oct 20;22:721. doi: 10.1186/s13063-021-05634-6 (PMC8527777; doi:10.1186/s13063-021-05634-6)
Supplement: Supplementary file 1 — Additional file 1: Table S1. The supplementary shows published data for the outcomes for interventions for cerebral malaria. Outcome for intravenous quinine alone was 23% to 26% while combines intravenous quinine and artesunate is 12.5%. Sinclair D, Donegan S, Isba R, Lalloo DG: Artesunate versus quinine for treating severe malaria. The Cochrane Library. 2012, 10.1002/14651858.CD005967.pub4.a Hien TT, Arnold K, Vinh H, et al Comparison of artemisinin suppositories with intravenous artesunate and intravenous quinine in the treatment of cerebral malaria. Trans R Soc Trop Med Hyg 1992; 86: 582–83.b Bartoloni A, Tomasoni L, Bartalesi F, Sani S, Zammarchi L, Castelli F, et al. Combined intravenous treatment with artesunate and quinine for severe malaria in Italy. Am J Trop Med Hyg 2010; 83: 274–76.c [file 13063_2021_5634_MOESM1_ESM.docx]

**Supplementary Table 1**

| **Study** | **Sample size** | **IV Artesunate** | **Artemisinin suppositories** | **IV Quinine** | **IV Quinine and artesunate** |
| --- | --- | --- | --- | --- | --- |
| Artesunate versus quinine for treating severe malaria.^a^ | 292 subgroup analysis | 10.1% |  | 23.0% |  |
| Comparison of Artemisinin suppositories with intravenous artesunate and intravenous quinine in the treatment of cerebral malaria.^b^ | 79 | 16.5% | 27.8% | 26.7% |  |
| Combined intravenous treatment with artesunate and quinine for severe malaria in Italy.^c^ | Case series 8 patients |  |  |  | 12.5% |

*The supplementary shows published data for the outcomes for interventions for cerebral malaria. Outcome for intravenous quinine alone was 23% to 26% while combines intravenous quinine and artesunate is 12.5%.*

Sinclair D, Donegan S, Isba R, Lalloo DG: Artesunate versus quinine for treating severe malaria. The Cochrane Library. 2012, 10.1002/14651858.CD005967.pub4.^a^ Hien TT, Arnold K, Vinh H, et al Comparison of artemisinin suppositories with intravenous artesunate and intravenous quinine in the treatment of cerebral malaria. Trans R Soc Trop Med Hyg 1992; 86: 582–83.^b^ Bartoloni A, Tomasoni L, Bartalesi F, Sani S, Zammarchi L, Castelli F, et al. Combined intravenous treatment with artesunate and quinine for severe malaria in Italy. Am J Trop Med Hyg 2010; 83: 274–76.^c^
